# Retention in care and viral suppression after same‐day ART initiation: One‐year outcomes of the SLATE I and II individually randomized clinical trials in South Africa

**DOI:** 10.1002/jia2.25825

**Published:** 2021-10-06

**Authors:** Mhairi Maskew, Alana T. Brennan, Willem D. F. Venter, Matthew P. Fox, Lungisile Vezi, Sydney Rosen

**Affiliations:** ^1^ Health Economics and Epidemiology Research Office Department of Internal Medicine School of Clinical Medicine Faculty of Health Sciences University of the Witwatersrand Johannesburg South Africa; ^2^ Department of Global Health Boston University School of Public Health Boston Massachusetts USA; ^3^ Department of Epidemiology Boston University School of Public Health Boston Massachusetts USA; ^4^ Ezintsha Department of Internal Medicine School of Clinical Medicine Faculty of Health Sciences University of the Witwatersrand Johannesburg South Africa

**Keywords:** implementation science, randomized trial, retention, same‐day ART, South Africa, viral suppression

## Abstract

**Introduction:**

Same‐day initiation (SDI) of antiretroviral therapy (ART) for HIV consistently increases ART uptake, but concerns remain about higher attrition from care after initiation. We analysed 12‐month retention in the SLATE SDI trials.

**Methods:**

SLATE I (Simplified Algorithms for Treatment Eligibility I, enrolment 06 March–28 July 2017) and SLATE II (enrolment 14 March–18 September 2018) were individually randomized trials at public outpatient clinics in Johannesburg that enrolled patients not yet on ART and administered the SLATE I or II algorithm. This included a symptom self‐report, medical history, brief physical examination and readiness questionnaire to assess the eligibility for SDI. The studies compared the offer of SDI using the SLATE algorithms to standard of care initiation procedures. ART uptake and early retention were previously reported. Using routine clinic records, we conducted a pooled analysis of retention in care and HIV viral suppression 14 months after study enrolment, a time point equivalent to 12 months potential on ART, with an additional month allowed on either end to initiate ART and to return for the 12‐month visit.

**Results and discussion:**

We enrolled 1193 study participants (standard arms, *n* = 599, 50%; intervention arms, *n* = 594, 50%) and analysed by originally assigned groups. By 14 months after enrolment, 50% of intervention arm patients and 46% of standard arm patients remained in care at the initiating site (crude risk difference 4% (95% confidence interval −1%‐10%); crude relative risk 1.10 (0.97–1.23), with similar viral suppression between arms. Observed attrition from care at site by 14 months was high in both study arms, but we found no evidence that the offer of SDI led to greater overall attrition or lower rates of viral suppression 1 year after starting ART and may have generated small improvements. SDI may have shifted some attrition from before to after dispensing of the first dose of medication.

**Conclusions:**

An offer of SDI of ART, following a carefully designed protocol to identify patients who are eligible and ready to start treatment, is not inherently associated with an overall increase in patient attrition from care and leads to similar rates of viral suppression.

**Trial registration:**

Clinicaltrials.gov NCT02891135, registered 01 September 2016. First participant enrolled 06 March 2017 in South Africa. Clinicaltrials.gov NCT03315013, registered 19 October 2017. First participant enrolled 14 March 2018.

## 1 INTRODUCTION

Since 2017, when the World Health Organization recommended ‘same‐day initiation’ (SDI) of antiretroviral therapy (ART) for people living with HIV who are ready for treatment on the day they test positive for HIV [1], many countries in sub‐Saharan Africa, including South Africa [2], have introduced the possibility of SDI into their national HIV programmes. To help guide decisions on exactly who should be eligible for SDI and how to implement it, we developed and evaluated two algorithms in South Africa. SLATE I (Simplified Algorithms for Treatment Eligibility I) [3] and SLATE II [4] were designed as simple, clinical algorithms that require no point‐of‐care laboratory testing and can be used by existing healthcare personnel to distinguish patients who can start ART that day, even if they have mild symptoms of illness, from those who require additional care prior to initiation.

While evidence from the SLATE trials [5, 6] and others [7, 8] demonstrates improved uptake of ART with SDI compared to standard care, concerns remain whether the benefits of SDI can be translated into improved retention once on treatment, or if instead attrition is simply shifted from before to soon after ART initiation, or even made worse by pressure that the expectation of SDI is perceived to place on patients [9, 10]. Overall attrition at 8 months was lower in the intervention arm in both trials [5, 6], but its timing differed. In SLATE I, roughly 1/3 of the attrition observed in the standard arm but more than half in the intervention arm occurred after patients initiated ART; in SLATE II, half of the attrition observed in the standard arm but nearly 3/4 of the attrition observed in the intervention arm occurred after initiation. A limitation of both studies was that the primary outcome — a combined indicator of initiation of ART ≤28 days and retention in care 8 months after study enrolment — was assessed at a time representing just 6 months on ART. Full 12‐month outcomes remain unclear. We present SLATE I and SLATE II retention and viral suppression outcomes 14 months after study enrolment (a time point equivalent to 12 months’ potential on ART, with an additional month allowed on either end to initiate ART and to return for the 12 month visit) to determine whether the differences between arms observed at 8 months persisted to 14 months.

## 2 METHODS

### 2.1 Study design

**Figure 1:**
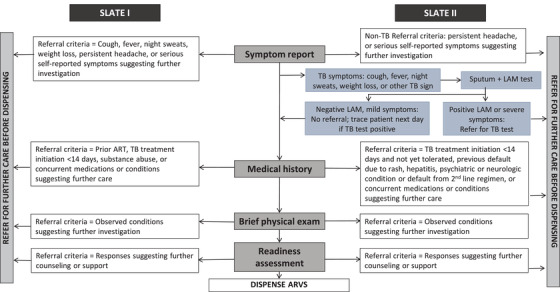
SLATE I and SLATE II algorithms. Abbreviations: ART, antiretroviral therapy; ARVs, antiretroviral medications; LAM, lipoaribomannan; SLATE, Simplified Algorithms for Treatment Eligibility; TB, tuberculosis

SLATE I and SLATE II were individually randomized, non‐blinded pragmatic evaluations to assess the effect of each SLATE algorithm on ART initiation and retention in care. Both studies have been described in detail elsewhere [3, 4, 5, 6, 11, 12]. Both algorithms consisted of four screening tools (Figure 1), each evaluating specific criteria for SDI: (1) symptom report, (2) medical history, (3) physical examination and (4) patient readiness assessment. Intervention arm patients found to be eligible on all four screening tools were offered initiation of ART on the day of study enrolment. Those ineligible on any of the screens were referred back to routine care for further services prior to ART initiation; clinics could still offer ART initiation that same day if they chose.

### 2.2 Study setting and population

Both studies were conducted in South Africa. The study sites were high‐volume, public‐sector primary care clinics in urban formal and informal settings around Johannesburg, South Africa. Study participants were enrolled from March to July 2017 for SLATE I and March to September 2018 for SLATE II. Care was provided according to the relevant South African National Department of Health ART guidelines during each study [13]. Non‐pregnant, HIV‐positive adults ≥ 18 years presenting at the study sites for HIV diagnosis or any type of HIV care but not yet on ART, including ART‐naïve patients and those returning to care after disengaging for >3 months, were eligible for the studies. Patients in both studies were initiated on the standard first‐line regimen of tenofovir, emtricitabine and efavirenz, dispensed in a combined once‐daily tablet.

### 2.3 Study procedures

Consented and enrolled study participants completed an interviewer‐administered questionnaire and were randomized 1:1 to either the intervention arm (SLATE algorithm) or standard arm (routine clinic procedures). After randomization, standard arm patients continued their clinic visits under standard of care. Patients randomized to the intervention arms were referred to a study nurse who administered the four SLATE algorithm screens and then dispensed ART directly to those patients eligible for SDI and referred back to the facility those patients requiring additional services prior to initiation of ART. Patients in both arms were followed passively, through medical record review, and had no further interaction with study staff. Patients who required clinical follow‐up after the study enrolment visit received routine care.

### 2.4 Study outcomes and data analysis

We report here the secondary outcomes of (1) initiation of ART within 28 days and retention on ART 14 months after study enrolment and (2) suppression of viral load (to <400 copies/ml) by 14 months after study enrolment. A patient was considered retained if we observed a clinic visit or laboratory test in the patient's medical record between 11 and 14 months after study enrolment. Fourteen months was selected to allow up to 1 month to initiate ART, 12 months of follow up after treatment initiation and up to 1 month to return for the 12‐month routine clinic visit. Patients with no evidence of a clinic visit or laboratory test during this period were assumed lost to follow up.

All follow‐up data were sourced from routinely collected medical records in TIER.Net [14], South Africa's national HIV monitoring system, and supplemented with routine electronic and paper records at the site and laboratory records from the National Health Laboratory System (NHLS). For this analysis, we pooled both study samples and conducted a crude analysis comparing the proportion of patients achieving each dichotomous outcome by study arm. We estimated crude risk ratios and crude risk differences and their corresponding 95% confidence intervals (CI) for each outcome, by intention‐to‐treat.

### 2.5 Ethics

The studies were approved by the Human Research Ethics Committee of the University of the Witwatersrand (Medical) and the institutional review board of Boston University Medical Campus. All study participants provided written informed consent.

## 3 RESULTS AND DISCUSSION

### 3.1 Summary of prior findings

Participants were enrolled between 06 March and 28 July 2017 for SLATE I and between 14 March and 18 September 2018 for SLATE II. A total of 1193 study participants in both studies were randomized to the standard arm (*n* = 599, 50%) or the intervention arm (*n* = 594, 50%) and analysed by originally assigned groups. The study population comprised predominantly women (63%) with a median age of 35 years (IQR 29–41) and median CD4 count of 293 cells/mm^3^ (133–487). No important imbalances with respect to the characteristics of the enrolled sample were noted.

In the combined sample of 1193, a total of 447/599 standard arm patients (74.6%) and 509/594 intervention arm patients (85.7%) initiated ART within 28 days of study enrolment. Achievement of the combined primary outcome of initiation ≤28 days and retention in care at 8 months was 321/599 (53.6%) and 381/594 (64.1%) for the standard and intervention arms, respectively, revealing very high rates of attrition from these sites — 46.4% and 35.9% — by 8 months after study enrolment.

### 3.2 Retention and suppression at 14‐month endpoint

In Table 1, we report outcomes at 14 months after study enrolment. Initiation ≤28 days and retention at 8 months are included in Table 1 for comparison. Separate results for each study are provided in Tables S1 and S2.

**Table 1:** Retention and viral suppression outcomes at 14 months after study enrolment by study arm in the SLATE I and SLATE II study populations (*n*= 1193)

Outcome	Standard arms (*n*=599)	Intervention arms (*n*=594)	Crude RD (95%CI)^a^	Crude RR (95% CI)^a^
Previously reported outcomes				
Initiated ART ≤ 28 days of study enrolment	447 (75%)	509 (86%)	11% (7–16%)	1.15 (1.08–1.22)
Initiated ART ≤ 28 days and retained in care 8 months after study enrolment	321 (54%)	381 (64%)	10% (5–16%)	1.20 (1.09–1.32)
Initiated ART ≤ 28 days and known to be virally suppressed by 8 months	184 (31%)	223 (38%)	7% (1–12%)	1.22 (1.04–1.43)
14‐month outcomes (retention)^b^				
Initiated ART ≤ 28 days and retained in care 14 months after study enrolment	275 (46%)	299 (50%)	4% (–1% to 10%)	1.10 (0.97–1.23)
Initiated ART ≤ 28 days, not retained 14 months after study enrolment	173 (29%)	210 (35%)	6% (1–12%)	1.22 (1.04–1.45)
Did not initiate ≤ 28 days	151 (25%)	85 (14%)	–11% (–15% to 6%)	0.57 (0.45–0.72)
14‐month outcomes (viral suppression)^c^				
Initiated ART ≤ 28 days and known to be virally suppressed by 14 months	163 (27%)	173 (29%)	2% (–3% to 7%)	1.07 (0.89–1.28)
Initiated ART ≤ 28 days and known to be virally unsuppressed by 14 months	15 (3%)	14 (2%)	0% (–2% to 2%)	0.94 (0.46–1.93)
No viral load test results found	97 (16%)	112 (19%)	3% (–2% to 8%)	1.16 (0.91–1.49)
Alternate 14‐month outcomes (viral suppression)^d^				
Initiated ART ≤ 28 days and known to be virally suppressed by 14 months	181 (30%)	199 (34%)	4% (–2% to 9%)	1.11 (0.94–1.31)
Initiated ART ≤ 28 days and known to be virally unsuppressed by 14 months	18 (3%)	18 (3%)	0% (–2% to 2%)	1.00 (0.53–1.91)
No viral load test results found	76 (13%)	82 (13%)	1% (–3% to 5%)	1.09 (0.81–1.46)
Alternate viral load outcomes^e^				
Any suppressed viral load after study enrolment	297 (50%)	320 (54%)	4% (–1% to 10%)	1.09 (0.97–1.21)
Any unsuppressed viral load result after study enrolment	37 (6%)	37 (6%)	38 (6%)	0% (–3% to 3%)
No viral load results observed	265 (44%)	236 (40%)	–4% (–10% to 1%)	0.90 (0.79–1.03)

^a^ Reference group: standard arm.

^b^ Per protocol outcome definition: Observed clinic visit or VL test between months 11–14 after study enrolment.

^c^ Per protocol outcome definition: Observed VL test between months 11–14 after study enrolment.

^d^ Alternate outcome definition: Observed VL test between months 9–14 after study enrolment.

^e^ Alternate outcome definition: Any viral load test result observed at any point up to 14 months after study enrolment regardless of whether the patient initiated ART or not.

Abbreviations: ART, antiretroviral therapy; CI, confidence interval; RD, risk difference; RR, risk ratio.

Retention in care at the study sites by 14 months was poor in both study arms. The numerical advantage seen in the intervention arms of the trials at the 8‐month primary endpoints, however, persisted through 14 months, when 50% of intervention arm patients remained in care compared to 46% of standard arm patients. The timing of attrition from care was also consistent: more standard arm patients (25% standard vs. 14% intervention) failed to initiate ART within 28 days, while more intervention arm patients were lost after initiation (35% intervention vs. 29% standard).

We traced viral load tests results between 9 and 14 months after enrolment for 365 (64%) of the 574 study patients retained in care through 14 months. Viral suppression was similar between the arms (30% vs. 34% for standard and intervention arm patients, respectively). Viral suppression rates were high among those with a test result, with only 3% observed with an unsuppressed HIV viral load result in either arm. Table 1 presents two alternate outcome definitions for viral suppression: (1) observed viral load tests between 9 and 14 months after study enrolment to allow for the possibility that high rates of missing test results were due to the 12‐month viral load test being done earlier; and (2) observed viral load tests done at any point up to 14 months after enrolment to measure differences in viral load suppression independent of ART initiation. Sensitivity analyses using different assumptions about missing viral loads are reported in Table S3.

Our results suggested that there may be differences between men and women in both outcomes, as shown in Table S4.

We also traced ART initiation records for patients who did not initiate within 28 days. In the combined intervention arms, 5% of participants initiated ART between 28 and 90 days after study enrolment, and one patient initiated after 90 days. Among standard arm patients, we observed an additional 7% and 3% of patients initiating 28–90 days and >90 days after enrolment, respectively. Among those with an observed ART start date, the median time to initiation in the intervention arms was 0 days (IQR 0–0; range 0–136 days post study enrolment), while for the standard arms, the median was 6 days (IQR 0–13 days; range 0–191 days post study enrolment). We note that patients who initiated ART during the study period but not ≤6 months after study enrolment may not have reached the scheduled date for their first viral load test by 14 months; these patients would have been among those missing viral load tests in Table 1.

In this extended analysis of the SLATE I and SLATE II trial data from South Africa, we found that attrition from care at site by 14 months was high in both studies and both arms, but we found no evidence that the timing of ART initiation led to greater overall attrition 1 year after patients had the opportunity to start ART. At the same time, it does appear that the offer of SDI shifted some attrition from before to after dispensing of the first dose of medication. For some intervention arm patients — those who were not in fact ready to start ART — it may have been easier to accept the offer of SDI and drop out of care afterwards than to refuse the offer when face‐to‐face with the care provider. We interpret this result to mean not that SDI ‘causes’ post‐initiation attrition from care, but rather that there is a certain proportion of patients who will drop out of care no matter how it is delivered, and an offer of SDI will not change this fact [15]. What SDI can do, as we stated in an earlier publication, is ‘to prompt those who do make it to the clinic at least once to give ART a try, rather than being sent away empty‐handed’ [5]. The potential for resistance development among patients disengaging from care early after SDI, though still important to monitor, is far less likely in an era of widespread use of highly potent integrase inhibitors with high barriers to resistance.

Recent observational studies of routine ART initiation have reported higher loss to follow‐up among patients who start ART on the same day than among those who do not [16, 17, 18]. We speculate that there are two main explanations for the discrepancy between our results and these studies’ findings. First, it is likely that the patients offered and accepting SDI in routine service delivery differ from the ART‐eligible patient population as a whole. Providers may offer SDI to patients whom they fear will not return for a second initiation visit, for example, but these may also be patients who are at higher risk of post‐initiation loss to follow‐up. Second, the SLATE algorithms included more than just the offer of SDI. Intervention arm patients participated in a structured preparation process implemented by trained study staff who may have been more successful in motivating patients to remain in care. The quality of the ART initiation process may thus be an important predictor of outcomes, along with the timing.

Our previous reports noted a number of limitations of the SLATE trials [5, 6]. The limitation most likely to have affected these results was our reliance on routinely collected data and the absence of unique identification numbers in the South African health system. This meant that we could not ascertain the true outcomes of those who appeared to be lost to follow‐up, some of whom likely remained on or re‐started ART at other facilities. We have no reason to suspect that this limitation would have affected our study arms differentially, however. While rates of loss to follow‐up in our study were high, they were not outside the bounds reported by other studies [19, 20]. Loss to follow‐up and treatment interruptions during the first year after starting ART are common throughout the sub‐Saharan region and are a critically important challenge for the success of national ART programmes [21].

## 4 CONCLUSIONS

We conclude that an offer of SDI of ART, following a carefully designed protocol to identify patients who are eligible and ready to start treatment, is not inherently associated with an overall increase in patient attrition from care.

## COMPETING INTERESTS

WDFV sits on antiretroviral initiation guideline committees both local and international, has accepted speaking honoraria from multiple manufacturers of antiretrovirals and is on several of their advisory boards. The remaining authors declare that they have no competing interests.

## AUTHORS’ CONTRIBUTIONS

MM, ATB, MPF, WDFV and SR conceptualized the study and designed the protocol. LV, ATB and MM collected and curated the data. MM, ATB and MPF analysed the data. MM and SR drafted the manuscript. All authors reviewed and approved the manuscript.

## Supporting information


**Table S1**. Results for SLATE II.
**Table S2**. Results for SLATE I.
**Table S3**. Results for sensitivity analyses for missing viral load data (per protocol definition).
**Table S4**. Retention and viral suppression outcomes at 14 months after study enrolment by study arm and sex in the SLATE I and SLATE II study populations (*n* = 1193).
