# Supplementary material for: Retention in care and viral suppression after same‐day ART initiation: One‐year outcomes of the SLATE I and II individually randomized clinical trials in South Africa
Source: J Int AIDS Soc. 2021 Oct 6;24(10):e25825. doi: 10.1002/jia2.25825 (PMC8694178; doi:10.1002/jia2.25825)
Supplement: Supplementary file 1 — Table S1. Results for SLATE II. Table S2. Results for SLATE I. Table S3. Results for sensitivity analyses for missing viral load data (per protocol definition). Table S4. Retention and viral suppression outcomes at 14 months after study enrolment by study arm and sex in the SLATE I and SLATE II study populations (n = 1193). [file JIA2-24-e25825-s001.docx]

**SUPPLEMENTARY MATERIALS**

**Supplementary table 1: Results for SLATE I**

| **Outcome** | **Standard arms (n=302)** | **Intervention arms (n=298)** | **Crude RD (95%CI)†** | **Crude RR (95% CI)†** |
| --- | --- | --- | --- | --- |
| **Previously reported outcomes** |  |  |  |  |
| Initiated ART ≤ 28 days of study enrolment | 204 (68%) | 232 (78%) | 10% (3 to 17%) | 1.15 (1.04 to 1.27) |
| Initiated ART ≤ 28 days and retained in care 8 months after study enrolment | 146 (48%) | 161 (54%) | 6% (-2% to 14%) | 1.12 (0.96 to 1.31) |
| Initiated ART ≤ 28 days and known to be virally suppressed by 8 months | 90 (30%) | 93 (31%) | 1% (-6% to 9%) | 1.05 (0.82 to 1.33) |
| **14-month outcomes (retention)*** |  |  |  |  |
| Initiated ART ≤ 28 days and retained in care 14 months after study enrolment | 153 (51%) | 149 (50%) | -1% (-9% to 7%) | 0.99 (0.84-1.16) |
| Initiated ART ≤ 28 days, not retained 14 months after study enrolment | 51 (17%) | 83 (28%) | 11% (4% to 18%) | 1.65 (1.21-2.25) |
| Did not initiate ≤ 28 days | 98 (32%) | 66 (22%) | -10% (-17 to -3%) | 0.68 (0.52-0.89) |
| **14-month outcomes (viral suppression)**** |  |  |  |  |
| Initiated ART ≤ 28 days and known to be retained and virally suppressed by 14 months | 65 (22%) | 68 (23%) | 1% (-5% to 8%) | 1.06 (0.79-1.43) |
| Initiated ART ≤ 28 days and known to be virally unsuppressed by 14 months | 8 (3%) | 2 (1%) | -2% (-4% to 0%) | 0.25 (0.05-1.18) |
| No viral load test results found | 80 (26%) | 79 (26%) | 0% (-7% to 7%) | 1.00 (0.77-1.31) |

*Observed clinic visit or VL test between months 11-14 after study enrolment

**Observed VL test between months 11-14 after study enrolment

**Supplementary table 2: Results for SLATE II**

| **Outcome** | **Standard arms (n=297)** | **Intervention arms (n=296)** | **Crude RD (95%CI)†** | **Crude RR (95% CI)†** |
| --- | --- | --- | --- | --- |
| **Previously reported outcomes** |  |  |  |  |
| Initiated ART ≤ 28 days of study enrolment | 243 (82%) | 277 (94%) | 12% (7% to17%) | 1.14 (1.08–1.22) |
| Initiated ART ≤ 28 days and retained in care 8 months after study enrolment | 175 (59%) | 220 (74%) | 15% (8% to 23%) | 1.26 (1.12–1.42) |
| Initiated ART ≤ 28 days and known to be virally suppressed by 8 months | 94 (32%) | 130 (44%) | 12% (5% to 20%) | 1.39 (1.12–1.71) |
| **14-month outcomes (retention)*** |  |  |  |  |
| Initiated ART ≤ 28 days and retained in care 14 months after study enrolment | 166 (56%) | 168 (57%) | 1% (-7% to 9%) | 1.02 (0.88-1.17) |
| Initiated ART ≤ 28 days, not retained 14 months after study enrolment | 77 (26%) | 109 (37%) | 11% (3% to 18%) | 1.42 (1.11-1.81) |
| Did not initiate ≤ 28 days | 54 (18%) | 19 (6%) | 12% (6% to 17%) | 0.35 (0.21-0.58) |
| **14-month outcomes (viral suppression)**** |  |  |  |  |
| Initiated ART ≤ 28 days and known to be retained and virally suppressed by 14 months | 111 (37%) | 115 (39%) | 2% (-6% to 9%) | 1.04 (0.85-1.28) |
| Initiated ART ≤ 28 days and known to be virally unsuppressed by 14 months | 11 (4%) | 13 (4%) | 0% (-2% to 4%) | 1.19 (0.54-2.60) |
| No viral load test results found | 44 (15%) | 40 (14%) | 1% (-7% to 4%) | 0.91 (0.61-1.36) |

*Observed clinic visit or VL test between months 11-14 after study enrolment

**Observed VL test between months 11-14 after study enrolment

**Supplementary table 3: Results for sensitivity analyses for missing viral load data (per protocol definition)**

| **Outcome** | **Standard arms (n=599)** | **Intervention arms (n=594)** | **Crude RD (95%CI)†** | **Crude RR (95% CI)†** |
| --- | --- | --- | --- | --- |
| **14-month outcomes (viral suppression) – Original analysis** | | | | |
| Initiated ART ≤ 28 days and known to be retained and virally suppressed by 14 months | 163 (27%) | 173 (29%) | 2% (-3 to 7%) | 1.07 (0.89-1.28) |
| Initiated ART ≤ 28 days and known to be virally unsuppressed by 14 months | 15 (3%) | 14 (2%) | 0% (-2 to 2%) | 0.94 (0.46-1.93) |
| No viral load test results found | 97 (16%) | 112 (19%) | 3% (-2 to 8%) | 1.16 (0.91-1.49) |
| **14-month outcomes (viral suppression) – sensitivity analysis assuming all missing tests were unsuppressed VL** | | | | |
| Initiated ART ≤ 28 days and known to be retained and virally suppressed by 14 months | 163 (27%) | 173 (29%) | 1% (-5% to 8%) | 1.06 (0.79-1.43) |
| Initiated ART ≤ 28 days and known to be virally unsuppressed by 14 months | 112 (19%) | 126 (21%) | 2% (-2% to 7%) | 1.13 (0.90-1.42) |
| **14-month outcomes (viral suppression) – sensitivity analysis assuming all missing tests were suppressed VL** | | | | |
| Initiated ART ≤ 28 days and known to be retained and virally suppressed by 14 months | 260 (43%) | 285 (48%) | 5% (-1% to 10%) | 1.11 (0.98-1.25) |
| Initiated ART ≤ 28 days and known to be virally unsuppressed by 14 months | 15 (3%) | 14 (2%) | -2% (-4% to 0%) | 0.25 (0.05-1.18) |
| **14-month outcomes (viral suppression) – sensitivity analysis assuming all standard arm missing VL tests were suppressed and all intervention arm missing VL tests were unsuppressed** | | | | |
| Initiated ART ≤ 28 days and known to be retained and virally suppressed by 14 months | 260 (43%) | 173 (29%) | -14% (-20% to -9%) | 0.67 (0.57-0.78) |
| Initiated ART ≤ 28 days and known to be virally unsuppressed by 14 months | 15 (3%) | 126 (21%) | 18% (15% to 22%) | 8.47 (5.02-14.29) |
| **14-month outcomes (viral suppression) – sensitivity analysis assuming all standard arm missing VL tests were unsuppressed and all intervention are missing VL tests were suppressed** | | | | |
| Initiated ART ≤ 28 days and known to be retained and virally suppressed by 14 months | 163 (27%) | 285 (48%) | 21% (15% to 26%) | 1.76 (1.51-2.06) |
| Initiated ART ≤ 28 days and known to be virally unsuppressed by 14 months | 112 (19%) | 14 (2%) | -16% (-20% to -13%) | 0.13 (0.07-0.22) |

**Supplementary table 4: Retention and viral suppression outcomes at 14 months after study enrolment by study arm and sex in the SLATE I and SLATE II study populations (n= 1193)**

| **Stratification variables** | **Initiated ART ≤ 28 days and retained in care 14 months after study enrolment** | | | | **Initiated ART ≤ 28 days and known to be virally suppressed by 14 months** | | | |
| --- | --- | --- | --- | --- | --- | --- | --- | --- |
|  | **Standard arm (n=599)** | **Intervention arm (n=594)** | **Crude RD**  **(95% CI)** | **Crude RR**  **(95% CI)** | **Standard arm (n=599)** | **Intervention arm (n=594)** | **Crude RD**  **(95% CI)** | **Crude RR**  **(95% CI)** |
| All participants | 275/599 (46%) | 299/594 (50%) | 4%  (-1 to 10%) | 1.10  (0.97-1.23) | 163/599 (27%) | 173/594 (29%) | 2%  (-3 to 7%) | 1.07  (0.89-1.28) |
| Men | 105/225 (47%) | 103/216 (48%) | 1%  (-8 to 10%) | 1.02  (0.84-1.25) | 70/225 (31%) | 59/216  (27%) | -4%  (-12 to 5%) | 0.88  (0.66-1.18) |
| Women | 170/374 (45%) | 196/378 (52%) | 6%  (-1 to 14%) | 1.14  (0.98-1.32) | 93/374 (25%) | 114/378 (30%) | 5%  (-1 to 12%) | 1.21  (0.96-1.53) |
